# Supplementary material for: Restraint of Human Skin Fibroblast Motility, Migration, and Cell Surface Actin Dynamics, by Pannexin 1 and P2X7 Receptor Signaling
Source: Int J Mol Sci. 2021 Jan 22;22(3):1069. doi: 10.3390/ijms22031069 (PMC7865282; doi:10.3390/ijms22031069)
Supplement: Supplementary file 1 [file ijms-22-01069-s001.pdf]

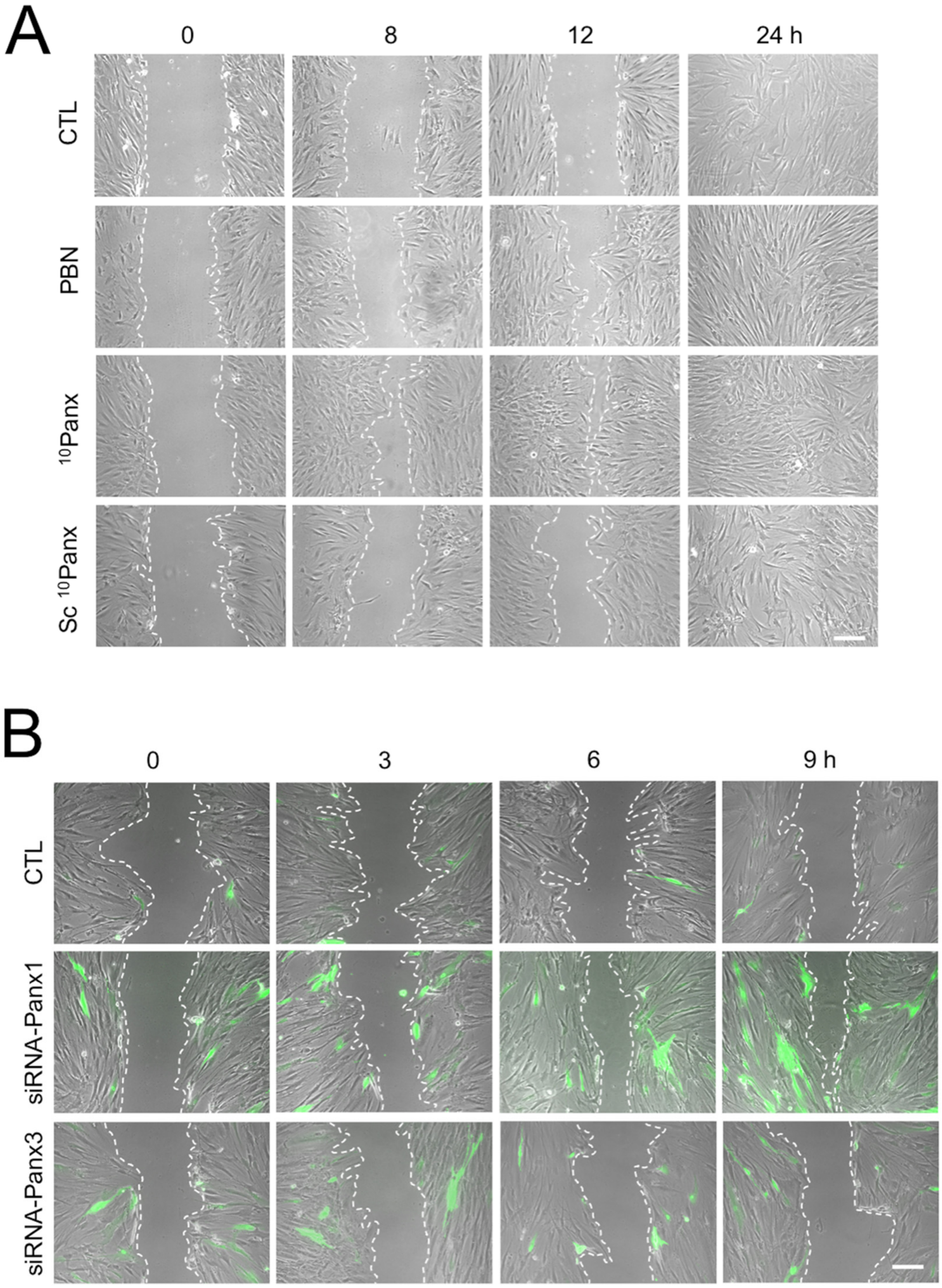

Figure S1. Inhibition or knockdown of Panx1 channels accelerates wound closure in HDF cultures. (**A**) Time-lapse representative images from in vitro wound closure of HDF cultures in each experimental condition at 0, 8, 12, and 24 h after scratch of the cultures. The dotted lines define the area lacking cells. Bar: 200 μm. (**B**) Superimposition of phase-contrast and fluorescence images of experiments shown in Figure 2A. HDF transfected cells with siRNAs against Panx1, Panx3 or control plasmid (CTL) were identified by expression of GFP. The dotted lines define the scratch area lacking cells. Bar: 200 μm.

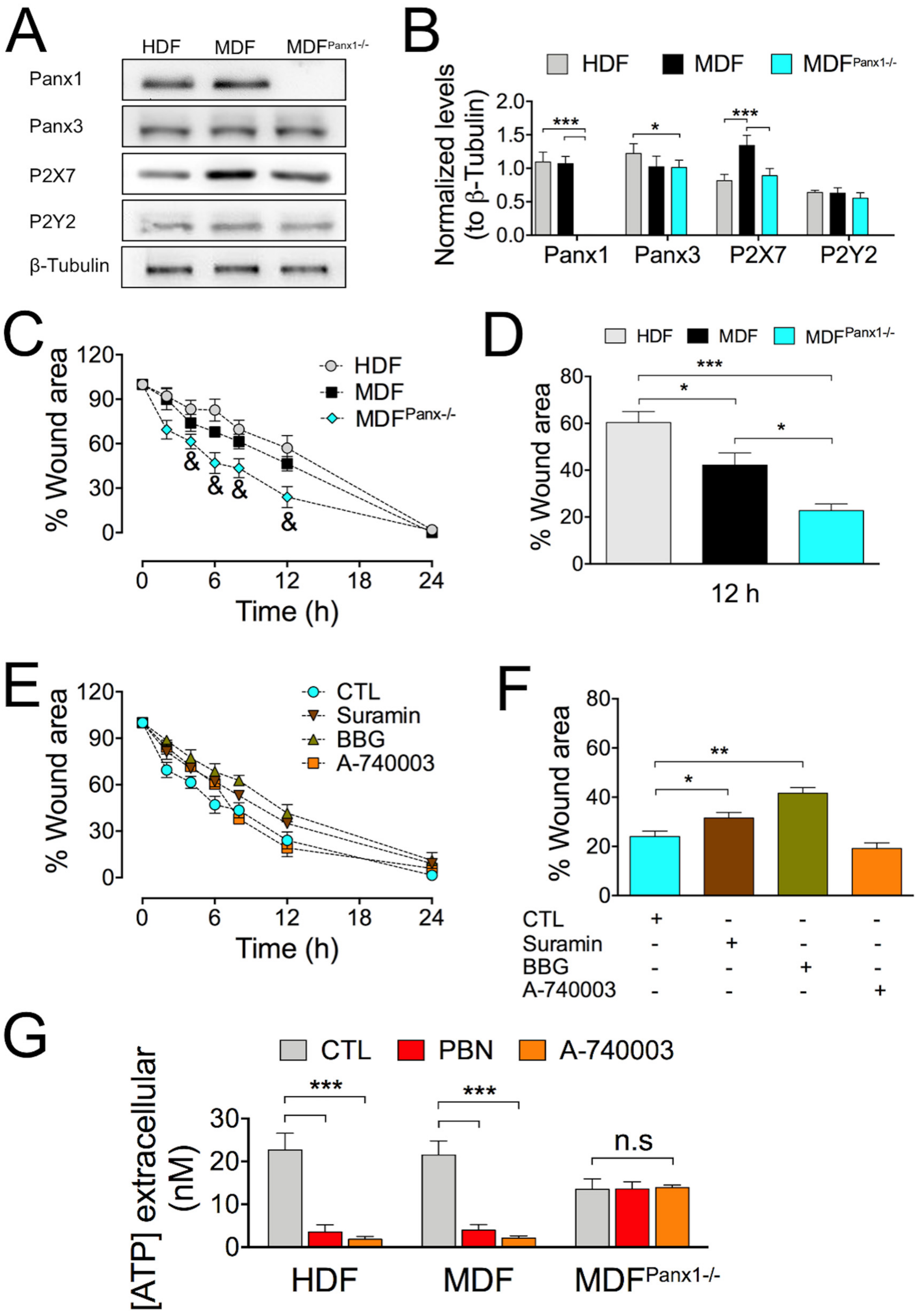

Figure S2. Inhibition of P2X7R did not accelerate migration in dermal fibroblast from Panx1-KO mice. (**A**) Representative Western blot of Panx1, Panx3, P2X7R and P2Y2R from homogenates of HDFs, wild-type mouse fibroblasts (MDF) and Panx1-KO mice fibroblasts ($MDF^{Panx1-/-}$). β-tubulin

signal was used as loading control. (**B**) Graphs depicting the relative amount of Panx1, Panx3, P2X7 and P2Y2 normalized to β-tubulin. Data were analyzed by two-way ANOVA followed by Tukey's post-hoc test (p < 0.05). *** p < 0.001; * p < 0.05; n= 6 cultures. (**C**) Comparison of wound-healing assay between HDF, MDF and $MDF^{Panx1-/-}$. Data were analyzed by two-way ANOVA followed by Bonferroni's post-hoc test. #= *** p < 0.001; &= ** p < 0.01 n=10 experiments. (**D**) Percentage of wound reduction after 12 h of making the wound in the respective cell cultures. One-way ANOVA followed by Tukey's post-hoc test. * p = 0.0207; *** p < 0.001; n = 10 experimental replicas. (**E**) Effect purinergic receptors blockers on wound healing assay in $MDF^{Panx1-/}$cultures; n = 10. (**F**) Percentage of wound reduction after 12 h of making the wound for the treatments shown in E. One-way ANOVA followed by Tukey's post-hoc test. * p = 0.0232; ** p < 0.01. (**G**) Measurements of extracellular ATP in cultures of HDFs, MDF and $MDF^{Panx1-/-}$ without (CTL) or with treatment with PBN or A-740003 after 12 h of wound healing assay. Two-way ANOVA followed by Bonferroni's post-hoc test; * p < 0.05; *** p < 0.001; n.s., not significant; n = 10 experiments. All data are shown as mean ± SEM from at least four different cultures.

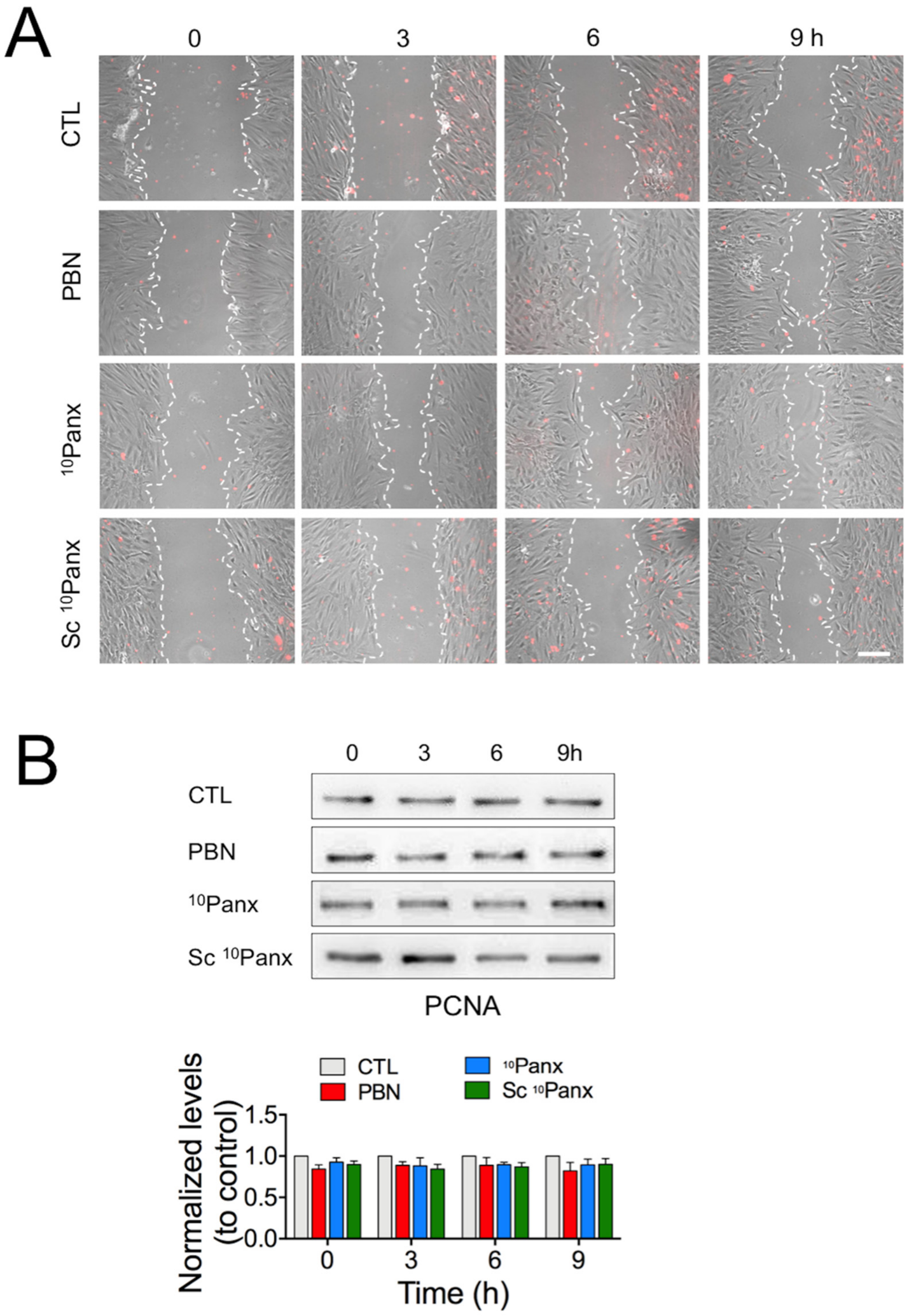

Figure S3. Activity of Panx1 channels during wound healing assay in HDF primary cultures. **(A)** Superimposition of phase-contrast and fluorescence images of experiments shown in Figure 3A. The dotted lines define the scratch or wound area. Bar: 200 μm. **(B-C)** Analysis of PCNA protein expression at different times of wound healing assay from HDF cultured under control condition, or with treatment with Panx1 blockers (PBN or ^{10}Panx1) or control peptide (Sc ^{10}Panx1). Upper panels show representative Western blot of PCNA for each time and experimental conditions. Graph

shows levels of PCNA normalized to levels found in control condition for each time and treatment. Two-way ANOVA followed by Bonferroni's post-hoc test. Not statistical significant was observed between treatments; $n = 10$. All data are shown as mean ± SEM from at least four different cultures.

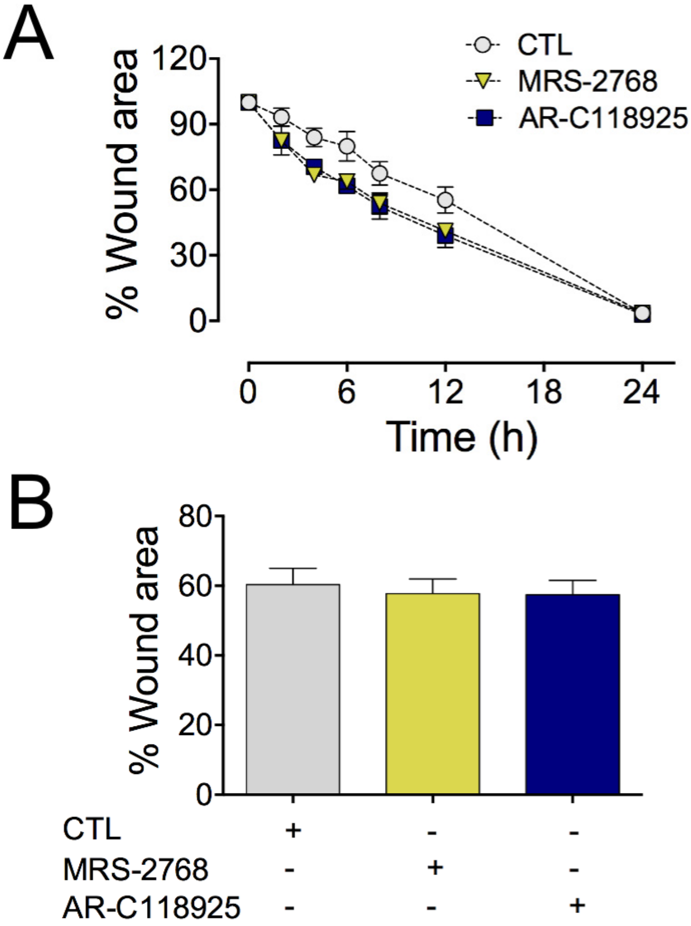

Figure S4. (A-B) The migration rate in wound healing assay of HDF is not affected by treatment with selective agonist or antagonist of P2Y2R, 10 μM MRS-2768 or 10 μM AR-C118925, respectively**.**. Comparison between migration rates after 12 h of inducing wound. One-way ANOVA followed by Tukey's post-hoc test. Values were not statistical different; n = 10 experiments. All data are shown as mean ± SEM from at least four different cultures.

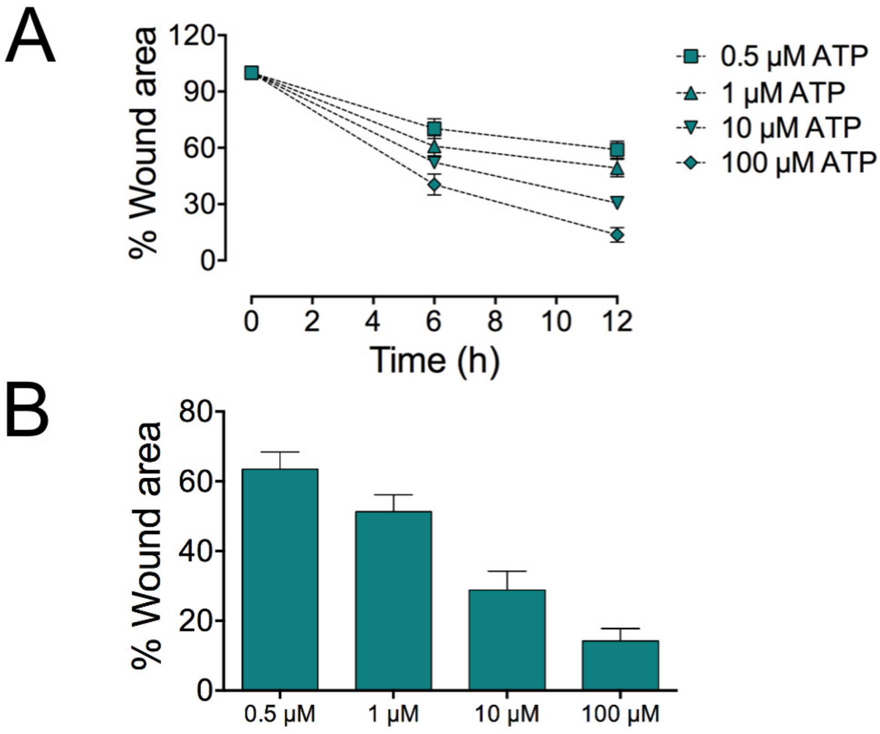

Figure S5. Higher concentrations of extracellular ATP accelerate migration. **(A)** Evaluation of the migration rate of HDF using wound-healing assay as a function of extracellular ATP concentration. **(B)** Comparison between migration rates after 12 h of inducing wound. *n* = 10 experiments. All data are shown as mean ± SEM from at least four different cultures.
